# Shuni Virus in Wildlife and Nonequine Domestic Animals, South Africa

**DOI:** 10.3201/eid2607.190770

**Published:** 2020-07

**Authors:** Jumari Steyn, Pebetsi Motlou, Charmaine van Eeden, Marthi Pretorius, Voula I. Stivaktas, June Williams, Louwtjie P. Snyman, Peter E. Buss, Brianna Beechler, Anna Jolles, Eva Perez-Martin, Jan G. Myburgh, Johan Steyl, Marietjie Venter

**Affiliations:** University of Pretoria Faculty of Health, Pretoria, South Africa (J. Steyn, P. Motlou, C. van Eeden, M. Pretorius, V.I. Stivaktas, M. Venter);; University of Pretoria Faculty of Veterinary Science, Pretoria (J. Williams, J.G. Myburgh, J. Steyl);; Durban Natural Science Museum, Durban, South Africa (L.P. Snyman);; South African National Parks, Kruger National Park, South Africa (P.E. Buss);; Oregon State University, Corvallis, Oregon, USA (B. Beechler, A. Jolles);; The Pirbright Institute, Woking, UK (E. Perez-Martin)

**Keywords:** Domestic animals, neurological disease, orthobunyavirus, RT-PCR, Shuni virus, South Africa, wildlife, viruses

## Abstract

We screened nonequine animals with unexplained neurologic signs or death in South Africa during 2010–2018 for Shuni virus (SHUV). SHUV was detected in 3.3% of wildlife, 1.1% of domestic, and 2.0% of avian species. Seropositivity was also demonstrated in wildlife. These results suggest a range of possible SHUV hosts in Africa.

Shuni virus (SHUV) (*Peribunyaviridae*: *Orthobunyavirus*) was isolated in the 1960s from livestock, *Culicoides* midges, and a febrile child in Nigeria (*1*,*2*). In South Africa, SHUV was identified as the causative agent of neurologic disease in horses (*3*); seropositivity was also demonstrated in 3.0% of veterinarians, suggesting human exposures (*4*). SHUV was subsequently identified in aborted livestock and cattle with neurologic disease in Israel, suggesting an extended range beyond Africa (*5*,*6*). We investigated other potential susceptible species in South Africa.

## The Study

During February 2010–September 2018, a total of 101 whole blood, 71 serum, and 476 tissue specimens from 608 nonequine domestic animals, wildlife, and birds (19 fetuses, 118 juvenile and 471 adults) with unexplained neurologic or febrile disease or sudden unexplained death from across South Africa were submitted to the Zoonotic Arbo and Respiratory Virus Program, Centre for Viral Zoonoses, University of Pretoria (Pretoria, South Africa) as part of a passive zoonotic arbovirus surveillance program. We extracted RNA under Biosafety Level (BSL) 3 conditions using the QIAamp viral RNA mini kit (blood) or the RNeasy mini kit (tissue) (QIAGEN, https://www.qiagen.com), according to the manufacturer’s recommendations. We screened all samples by a SHUV real-time reverse transcription PCR (rRT-PCR) (*7*) and a newly designed rRT-PCR targeting a conserved area of the S segment of the Simbu serogroup (Appendix). We confirmed PCR-positive samples by Sanger sequencing (Inqaba Biotech, https://www.inqababiotec.co.za) and phylogenetic analyses (Appendix). We also screened all specimens for West Nile (WNV), Wesselsbron (*8*), Middelburg (MIDV), Sindbis (*9*), and equine encephalosis viruses (*10*).

In addition, serum samples from African buffalo (*Syncerus caffer*) (n = 45) and white rhinoceros (*Ceratotherium simum*) (n = 48) from Kruger National Park were collected in March 2014 and June 2016, respectively, by South African National Parks and from wild Nile crocodiles (*Crocodylus niloticus*) (n = 34) from northern KwaZulu-Natal collected during 2009–2012 by the Faculty of Veterinary Sciences, University of Pretoria, as part of surveillance studies. We examined tissue samples from a SHUV PCR-confirmed positive buffalo (MVA43/10) microscopically under a light microscope using routinely prepared hematoxylin and eosin stained (*11*) histological sections at the Section of Pathology, Department of Paraclinical Sciences, Faculty of Veterinary Science, University of Pretoria. We subjected serum samples to an epitope-blocking ELISA (eb-ELISA) (*12*,*13*) with modifications to detect antibodies to SHUV (Appendix).

We calculated odds ratios (OR) and 95% CI in EpiInfo version 7.2.0.1 (https://www.cdc.gov/epiinfo/index.html). We excluded animals that were found dead, aborted, or stillborn from OR analysis.

We detected SHUV RNA in 15/608 (2.5%) animals tested from 10 different animal species: 12/361 (3.3%) wildlife, 2/196 (1.0%) nonequine domestic animals, and 1/51 avian species (2.0%) (Table 1). We detected SHUV in samples submitted from 2/62 (3.2%) white rhinoceroses, 2/50 (4.0%) sables, 1/15 (6.7%) warthog, 4/54 (7.4%) buffalo, 1/12 (8.3%) crocodiles, 1/5 (20.0%) giraffes, 1/4 (25.0%) springboks, 1/93 (1.1%) domestic bovids, and 1/10 (10.0%) alpacas (Table 1). We also detected SHUV in an exotic monal pheasant (1/13, 7.7%). Differential screening revealed co-infections with MIDV and WNV, suggesting that these arboviruses could co-circulate. A sable was also co-infected with *Theileria* sp. *sable* and *Theileria separate* (Table 1).

**Table 1:** Animals that tested positive for Shuni virus by real-time reverse transcription PCR, South Africa, 2010–2018*

Animal type	ID	No. positive/ no. tested	% Positive (95% CI)	Province where submitted	Positive specimen	Clinical signs	Co-infection
Domestic bovid	ZRU116/18	1/93	1.1 (0.0–3.1)	North West	Spleen	SUD	
White rhinoceros (*Ceratotherium simum*)	MVA11/10	2/62	3.2 (0.0–7.6)	Limpopo	CNS	Neurologic	MIDV
	ZRU137/18			Free State			
Sable (*Hippotragus niger*)	ZRU419/17	2/50	4.0 (0.0–9.4)	North West	Spleen	Hemorrhagic	Theileriosis
	ZRU121/18			Limpopo			
Warthog *(Phaecocherus africanus)*	MVA35/10	1/15	6.7 (0.0–19.3)	Limpopo	CNS	Neurologic, respiratory	
Buffalo (*Syncerus caffra*)	MVA43/10	4/54	7.4 (0.4–14.4)	Limpopo	CNS, whole blood	Neurologic, respiratory	
	ZRU77/18			Limpopo			
	ZRU97/18			Limpopo			
	ZRU166/18			Limpopo			
Monal (*Lophophorus impejanus*)	ZRU119/18	1/13	7.8 (0.0–22.2)	North West	CNS	SUD	
Crocodile (*Crocodylus niloticus*)	MVA08/10	1/12	8.3 (0.0–24.0)	Limpopo	CNS	Neurologic	
Alpaca (*Vicugna pacos*)	ZRU172/18	1/10	10.0 (0.0–28–6)	Western Cape	CNS	Neurologic, respiratory	
Giraffe (*Giraffa camelopardalis*)	ZRU87/18	1/5	20 (0.0–55.0)	North West	Whole blood	SUD	WNV
Springbok (*Antidorcus marsupialis*)†	ZRU261/17/3	1/4	25.0 (0.0–67.4)	Gauteng	Spleen	Neurologic	
Wildlife		12/361	3.3 (1.5–5.1)				
Domestic animals		2/196	1.1 (0.0–2.5)				
Avian		1/51	2.0 (0.0–5.8)				
Total		15/608	2.5 (1.2–3.7)				

*CNS, central nervous system; MIDV, Middelburg virus; SUD, sudden unexpected death; WNV, West Nile virus. †Cluster with Sango virus.

In 9/15 (60.0%, 95% CI 35.2%–84.8%) positive infections, we detected SHUV in the central nervous system (CNS) (Table 1), indicating passage across the blood–brain barrier, which suggests SHUV as the likely causal agent of the observed neurologic signs. This finding suggests that SHUV is not just an agent of subclinical infections or reproductive problems, such as abortion, as previously reported (*5*,*14*), but is also the likely etiology for neurologic disease in these species, as previously described for horses (*3*) and cattle (*6*). We did not detect SHUV RNA in aborted (n = 24) or stillborn (n = 16) animals. Eleven SHUV-positive animals showed neurologic signs (OR 1.8, 95% CI 0.2–14.4), with 2 animals also reported to be pyrexic (OR 2.0, 95% CI 0.4–9.4) or showing respiratory signs (OR 1.0, 95% CI 0.2–4.8) (Table 2). Three SHUV-positive animals were found dead (OR 1.8, 95% CI 0.5–6.4) (Table 2). Specific neurologic signs associated with SHUV infection included hind limb paresis progressing to quadriparesis with normal mentation (OR 6.7, 95% CI 2.0–22.5) (Table 2). 

**Table 2:** Clinical signs reported in wildlife, nonequine domestic animals, and birds upon submission to the Centre for Viral Zoonoses, South Africa, 2010–2018*

Sign	SHUV positive (%), n = 12	SHUV negative (%), n = 496	Odds ratio (95% CI)	p value†
Neurologic signs	11 (91.7)	415 (83.7)	1.8 (0.2–14.4)	0.9
Ataxia	2 (16.7)	102 (20.6)	0.8 (0.2–3.5)	1
Paralysis	3 (25.0)	61 (12.3)	2.3 (0.6–8.8)	0.4
Quadriparesis	8 (66.7)	112 (22.6)	6.7 (2.0–22.5)	<0.05
Recumbence	2 (16.7)	103 (20.8)	0.7 (0.2–3.4)	1
Pyrexia	2 (16.7)	44 (8.9)	2.0 (0.4–9.4)	0.7
Respiratory/dyspnea	2 (16.7)	79 (15.9)	1.0 (0.2–4.8)	1
Hemorrhage	1 (8.3)	10 (2.0)	4.3 (0.5–36.7)	0.6
Congenital deformities	0	7 (1.4)	Undefined	1
Outcomes	n = 15	n = 593		
SUD	3 (20.0)	74 (12.5)	1.8 (0.5–6.4)	0.6
Abortion	0	24 (4.1)	Undetermined	1
Stillbirth	0	16 (6.7)	Undetermined	1

*SHUV, Shuni virus; SUD, sudden unexpected death.  †p values <0.05 are regarded as significant.

Positivity of infection was highest in the North West (4/47, 8.5% of samples submitted from North West), followed by Limpopo Province (8/132, 6.1%) (Table 1; Appendix Figure 1). SHUV was detected only in 2010 (4/15, 26.7%), 2017 (2/15, 13.3%), and 2018 (9/15, 60.0%) despite continuous surveillance throughout the years, suggesting that outbreaks may be sporadic rather than annual. SHUV PCR positives were detected during April–September in each of the 3 years (Appendix Figure 2).

**Figure 1:**
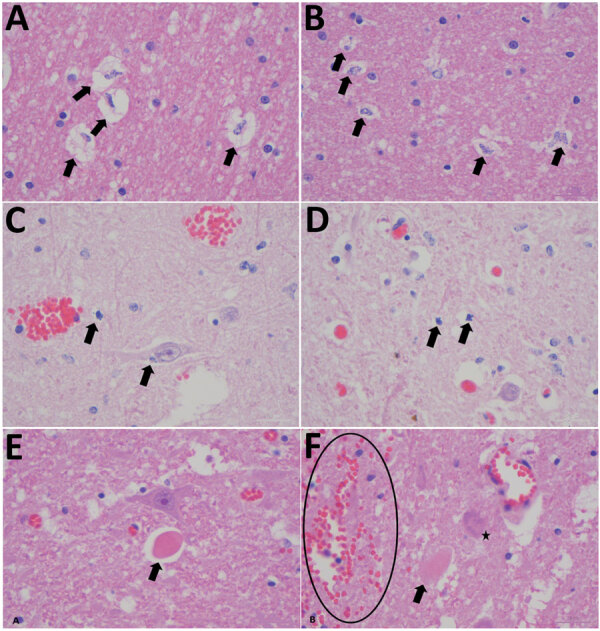
Histopathological changes in formalin-fixed brain tissue of a Shuni virus PCR-positive buffalo (MVA73/10) in South Africa that showed neurologic signs (original magnification 1000×). A, B) Cerebral white matter micro/astrogliosis and cytogenic edema (arrows). C, D) Glial (suspected oligodendroglia) apoptosis (arrows). E, F) Perineural hypereosinophilic bodies (arrows); perivascular and neuropil hemorrhage (circle); single-cell neuronal degeneration (chromatolysis) (star).

Necropsy examination on the buffalo showed no specific macroscopic lesions on histopathology examination of brain tissue (Figure 1). Pathological changes that could be detected in regions of the brain included mild white matter cerebro–cerebellar gliosis, especially microglial, associated with considerable glial apoptotic activity and occasional perivascular hemorrhage. In the spinal cord, occasional single neuronal necrosis (chromatolysis) and perineuronal hypereosinophilic bodies affecting the dorsal horns of the gray matter were distinctive. This finding seemed to be most severe in the lumbar spinal region. No evidence of demyelination or major immunological reaction was observed, apart from occasional perivascular lymphocytes. Development of appropriate antibodies for immunohistochemistry or probes for in situ hybridization may further describe the pathology of SHUV in animal tissue.

**Figure 2:**
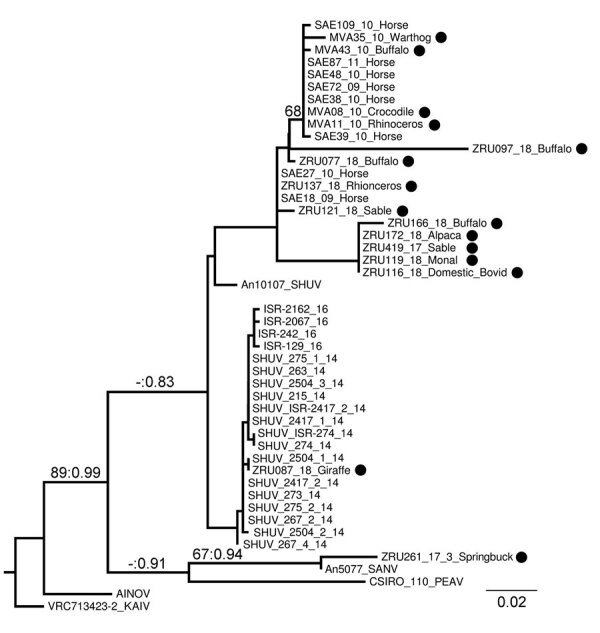
Phylogram of clade 1a, lineage I, of the Simbu serogroup (*15*) recovered from maximum-likelihood and Bayesian analyses of the small segment for SHUV isolates from wildlife and nonequine domestic animals, South Africa and reference sequences. Bootstrap values (maximum likelihood >60) and posterior probabilities (>0.8) are displayed on branches as support values. GenBank accession numbers for sequences from this study (black circles): MVA11_10_Rhinoceros, JQ726395; MVA08_10_Crocodile, JQ726396; MVA43_10_Buffalo, JQ726397; MVA35_10_Warthog, JQ726398; ZRU077_18_Buffalo, MK114084; ZRU121_18_Sable, MK114085; ZRU137_18_Rhinoceros, MK114086. GenBank accession numbers, virus types, and locations for reference sequences: An10107, AF362405, SHUV Nigeria; AINOV, M22011, Japan; VRC713423–2 KAIV, AF362394, India; SAE72_09_Horse, HQ610138, South Africa; SAE27_10_Horse, HQ610139, South Africa; SAE38_10_Horse, HQ610140, South Africa; SAE39_10_Horse, HQ610141, South Africa; SAE48_10_Horse, HQ610142, South Africa; SAE109_10_Horse, HQ610143, South Africa; SAE18_09_Horse, KC510272, South Africa; SAE87_11_Horse, KC525997, South Africa; Shuni_215_14, KP900859, Israel; Shuni_263_14, KP900860, Israel; Shuni_267_2_14, KP900861, Israel; Shuni_267_4_14, KP900862, Israel; Shuni_273_14, KP900865, Israel; Shuni_274_14, KP900867, Israel; Shuni_275_1_14, KP900869, Israel; Shuni_275_2_14, KP900871, Israel; Shuni_2417_1_14, KP900872, Israel; Shuni_2417_2_14, KP900875, Israel; Shuni_2504_1_14, KP900877, Israel; Shuni_2504_2_14, KP900878, Israel; Shuni_2504_3_14, KP900882, Israel; 2504_3_14, KU937313, Israel; SHUV_ISR-274_14, KT946779, Israel; SHUV_ISR-2417_2_14, KT946780, Israel; ISR-129_16, MF361846, Israel; ISR-242_16, MF361849, Israel; ISR-2067_16, MF361852, Israel; ISR-2162_16, MF361855, Israel; CSIRO 110, MH484320, Australia; An5077, AF362402, Nigeria. AINOV, ainovirus; KAIV, kaikalurvirus; PEAV, Peaton virus; SANV, Sango virus; SHUV, Shuni virus..

We used phylogenetic analyses on the small segment of the Simbu serogroup to verify the molecular results. All novel sequences from this study, with 1 exception, were closely related to SHUV strains identified in horses in South Africa (*3*) in clade 1a of lineage I within the Simbu serogroup (bootstopping: posterior probabilities = 89:0.99) (Figure 2). An isolate from a springbok (ZRU261_17_3) clustered with Sango virus (bootstopping: posterior probabilities = 67:0.94). P-distance analysis based on the partial small segment demonstrated few nucleotide differences between novel SHUV strains and reference strains (98.0%–100.0% identity). Wildlife specimens were submitted mostly from dead animals that were already undergoing postmortem cytolysis, inhibiting further genetic analysis and isolation of the virus. The use of a PCR designed to detect Simbu group/orthobunyavirus genus PCR rather than SHUV-specific PCR facilitated detection of these infections.

We detected antibodies to SHUV by an eb-ELISA in 3/44 (6.8%) African buffalo and 2/48 (4.2%) white rhinoceroses but none in crocodiles. SHUV-specific IgG was confirmed, using microtiter virus neutralization assay, in 1 buffalo and 1 rhinoceros. Two of 3 buffalo and 1 rhinoceros positive for SHUV epitope antibodies were negative by microtiter virus neutralization assay, suggesting that these antibodies may have been elicited in response to closely related orthobunyavirus. Confirmation for the third buffalo was not possible because of depleted serum.

## Conclusion

Our findings suggest that SHUV may have a wide host range, including several wildlife and domestic species, and should be included in the differential diagnosis of neurologic disease in animals. This study highlights the role of this virus as a potential emerging zoonotic pathogen in Africa that warrants increased surveillance and further investigation. Future epidemiologic studies would benefit from an increased sample size and more extensive serosurveys. Investigation of human infections may define SHUV’s importance as a zoonosis. The causative link between clinical manifestations in the various species and the evidence of SHUV infection must be regarded with caution because other possible infectious and noninfectious etiologies were not excluded by comprehensive investigations in all cases.

Appendix Additional information about the study of Shuni virus in wildlife and nonequine domestic animals, South Africa.
