# Supplementary material for: Shuni Virus in Wildlife and Nonequine Domestic Animals, South Africa
Source: Emerg Infect Dis. 2020 Jul;26(7):1521–5. doi: 10.3201/eid2607.190770 (PMC7323521; doi:10.3201/eid2607.190770)
Supplement: Appendix — Additional information about the study of Shuni virus in wildlife and nonequine domestic animals, South Africa. [file 19-0770-Techapp-s1.pdf]

Article DOI: https://doi.org/10.3201/eid2607.190770

Shuni Virus in Wildlife and Nonequine Domestic Animals, South Africa

Appendix

Methods and Validations

We designed and validated a new small (S) segment real-time reverse transcription PCR (rRT-PCR) assay using a more conserved region of the S segment of the Simbu virus serogroup, situated between genomic positions 152–304 (152 base pair [bp]) of SHUV sequence KC510272. We designed a group-specific primer set using viruses in the Simbu virus serogroup (Ortho(SimbuF152): 5′^{152}TAGAGTCTTCTTCCTCAAYCAGAAGAAGGCC′3^{184} and Ortho(SimbuR304): 5′^{276}GTYAMGGCAMTGTCTGGCACAGGATTTG′3^{304})) and a TaqMan probe (Ortho(Simbu-probe252)(5′^{252}TGGTTAATAACCATTTTCC′3^{270})) including the available SHUV strains. For the Simbu/orthobunyavirus-specific RT-PCR assay, a 50 µL reaction consisted of AgPath-ID 2x RT-PCR buffer (ThermoFisher Scientific, https://www.thermofisher.com), 10 µL RNA, 400 nM of the forward and reverse primers, 150 nM of the TaqMan probe, and a 25x RT-PCR Enzyme Mix (ThermoFisher Scientific). The 1-step real-time RT-PCR was performed on a ViiA 7 Real-Time PCR System (Applied Biosystems, https://www.thermofisher.com) with cycling conditions of 50°C for 30 min, 94°C for 2 min, and 45 cycles of 94°C for 15 sec and 58°C for 1 min. To validate the rtRT-PCR, a dilution series of RNA isolated from a culture control of SHUV strain SAE18/09 were created; the new Simbu/orthobunyavirus rRT-PCR ran in triplicate in parallel with the published SHUV-specific rRT-PCR (*1*). The published PCR could be detected up to 10^{-4} by melting curve analysis and up to 10^{-6} on an agarose gel. Each dilution could be detected by the Simbu/orthobunyavirus rRT-PCR at cycle threshold (Ct) values of 20 to 40 in triplicate up to 10^{-5} and with 2/3 replicates detected at 10^{-6} by the rRT-PCR at Ct values of 40 and visible on an agarose gel with the expected band sizes of 152 base pairs up to 10^{-6} (Appendix Table and Appendix Figure 3). Archived SHUV-positive clinical specimens were amplified in parallel with the SHUV-specific rRT-PCR (*1*) and the new Simbu/orthobunyavirus rtRT-PCR with amplification of 4/12 (33.3%)

samples with the SHUV specific rRT-PCR compared to 8/12 (66.7%) samples amplifying with the new Simbu/orthobunyavirus rtRT-PCR. This is likely the result of RNA degradation in the archived specimens with the new PCR being able to detect shorter fragments than the original PCR. Of a blinded panel of archived SHUV strains that could be detected with the SHUV nested rtRT-PCR (1), all could be detected by the Simbu/orthobunyavirus rRT-PCR, whereas no other arboviruses (WNV, MIDV, Sindbis, or Wesselsbron virus) could be detected. Three SHUV strains that could not be detected by the SHUV-specific PCR (*1*) was also detected in horses (reported elsewhere).

We edited and analyzed sequencing data using CLC Main Workbench 8 (https://www.qiagenbioinformatics.com). Multiple sequence alignments were done using multiple alignment using fast Fourier transform (MAFFT), v7 (http://mafft.cbrc.jp/alignment/server/index.html), and Mega 6.06 (Molecular Evolutionary Genetics Analysis software, https://www.megasoftware.net) was used to assemble concatenated sequences. Reference sequences from the Simbu serogroup were selected based on the gene segment and downloaded from GenBank. The best-fit model was estimated using jModelTest, v2.1.4 (https://github.com/ddarriba/jmodeltest2) and used to construct maximum likelihood trees using RAxML (https://cme.h-its.org/exelixis/web/software/raxml) with AutoMRE function. Bayesian analysis was performed in MrBayes version 3 (https://cme.h-its.org/exelixis/web/software/mrbayes). MrBayes 3 was used for Bayesian inference running 4 cold chains for 10,000,000 iterations, saving every 1000th tree. The bootstrap support of the maximum likelihood analysis for a concatenated (nucleotides [nt] = 490) phylogram of the "long" (nt = 338) (genomic position 305–643) and "short" (nt = 152) (genomic position 152–304) sequences was calculated from 650 replicates using the autoMRE bootstepping criterion in RaxML. GTR+G were applied to a partitioned dataset with bootstraps (bs) >60 viewed as supportive of the clustering (Figure 2). HKY+G and TIM2+G models were respectively applied to 2 unlinked partitions. All posterior probabilities (pb) >0.8 were viewed as strong support (Figure 2). Tracer v1.6 was employed for assessing distribution and effective sample size. Effective sample size (ESS) value was >>200. The number of nucleotide differences between sequences was determined using p-distance analysis with 1000 bootstrap replicates in MEGA 6.06. All SHUV nucleotide sequences >200 nt were submitted to GenBank prior to the study

(JQ726395–JQ726398 and MK114084–MK114086). Virus isolation was also attempted on positive tissue specimens, but it was not successful.

To perform the eb-ELISA, microtiter plates (CELLSTAR, Greiner Bio-One, Frickenhausen, Germany) were coated with 100 μL of a 1:100 dilution of SHUV-specific cell lysate antigen diluted in carbonate–bicarbonate buffer. SHUV antigen were produced using detergent basic buffer extraction of infected cell culture with reference strain SAE18/09 (*2,3*). Vero cells in 175 cm^2 flasks were infected and cultured until 80.0% cytopathic effects were observed. The cell suspension was clarified by centrifugation at 10,000 × *g* for 10 minutes at 4°C to pellet the cells. Cells were then washed in 0.01 M borate buffer saline (BBS) (Sigma-Aldrich, https://www.sigmaaldrich.com), pH 9.0. Cell pellet was resuspended in BBS and 10 μL 1.0% (vol/vol) Triton X-100 (Sigma-Aldrich). The suspension was sonicated for 10 minutes, placed on ice, and centrifuged at 10,000 × *g* for 5 min at 4°C, after which the supernatant was collected. Antigen coated plates were incubated overnight at 4°C in a humidity chamber. Two hundred microliters of blocking buffer (PBS containing 10.0% skim milk) was added to each well and incubated for 1 hour at 37°C. Test sera were diluted 1:4 in 2.0% skim milk and 100 μL was added. Plates were then incubated for 24 hours at 37°C. SHUV-specific hyperimmune mouse ascites fluid (MHIAF) polyclonal antibody (M12357A1, CDC, Fort Collins, USA) was diluted 1:200 in 2.0% skim milk and 100 μL was added to each well. Plates were then incubated for 1 hour at 37°C after which 100 μL of horseradish peroxidase (HRP)-conjugated rabbit anti-mouse IgG antibody (Bio-Rad Laboratories, https://www.bio-rad.com) was added at a dilution of 1:2000. Plates were incubated for 1 hour at 37°C. ABTS (2,2_azino-bis[3-ethylbenzthiazoline-6-sulfonic acid]) was added and plates were incubated for 30 minutes. After each incubation step, plates were washed 3 times with phosphate-buffered saline containing 0.05% Tween 20 (Sigma-Aldrich). The eb-ELISA was optimized using checkerboard titrations of different dilutions of the antigen and the antibody. Test sera were added at 1:4 dilution as recommended (*4,5*). The antibody binding was manually calculated, and expressed as percentage inhibition (PI) value, with a cutoff value of 40.0% PI. Positive reactions were confirmed with microtiter virus neutralization assay (micro-VNT) as described, using a 10^3 tissue-culture infective dose of SHUV culture (SAE18/09, passage 6) (*2*). A limitation to the validation of the eb-ELISA was the availability of known SHUV-positive animals other than horses. The eb-ELISA was therefore

optimized and validated using horse sera. Results of this study allows for further validation of the assay using wildlife sera.

References

1. van Eeden C, Zaayman D, Venter M. A sensitive nested real-time RT-PCR for the detection of Shuni virus. J Virol Methods. 2014;195:100–5.

2. van Eeden C, Swanepoel R, Venter M. Antibodies against West Nile and Shuni viruses in veterinarians, South Africa. Emerg Infect Dis. 2014;20:1409–11.

3. Ksiazek TG, West CP, Rollin PE, Jahrling PB, Peters CJ. ELISA for the detection of antibodies to Ebola viruses. J Infect Dis. 1999;179(Suppl 1):S192–8.

4. Blitvich BJ, Marlenee NL, Hall RA, Calisher CH, Bowen RA, Roehrig JT, et al. Epitope-blocking enzyme-linked immunosorbent assays for the detection of serum antibodies to West Nile virus in multiple avian species. J Clin Microbiol. 2003;41:1041–7.

5. Blitvich BJ, Bowen RA, Marlenee NL, Hall RA, Bunning ML, Beaty BJ. Epitope-blocking enzyme-linked immunosorbent assays for detection of West Nile virus antibodies in domestic mammals. J Clin Microbiol. 2003;41:2676–9.

Appendix Table. Summary of the TaqMan Simbu/orthubunyavirus genus specific real-time RT-PCR assay showing the cycle threshold values, mean, and standard deviation.

Dilution	Ct values (in triplicate)			Mean	Standard deviation
10^{0}	16.97	18.28	17.26	17.50	0.69
10^{-1}	20.96	20.94	20.82	20.91	0.08
10^{-2}	24.65	24.83	24.8	24.76	0.10
10^{-3}	28.54	28.58	28.65	28.59	0.56
10^{-4}	32.35	32.2	31.1	31.88	0.69
10^{-5}	36.57	36.17	36.21	36.32	0.22
10^{-6}	40.16	40.08	40.11	40.12	0.06

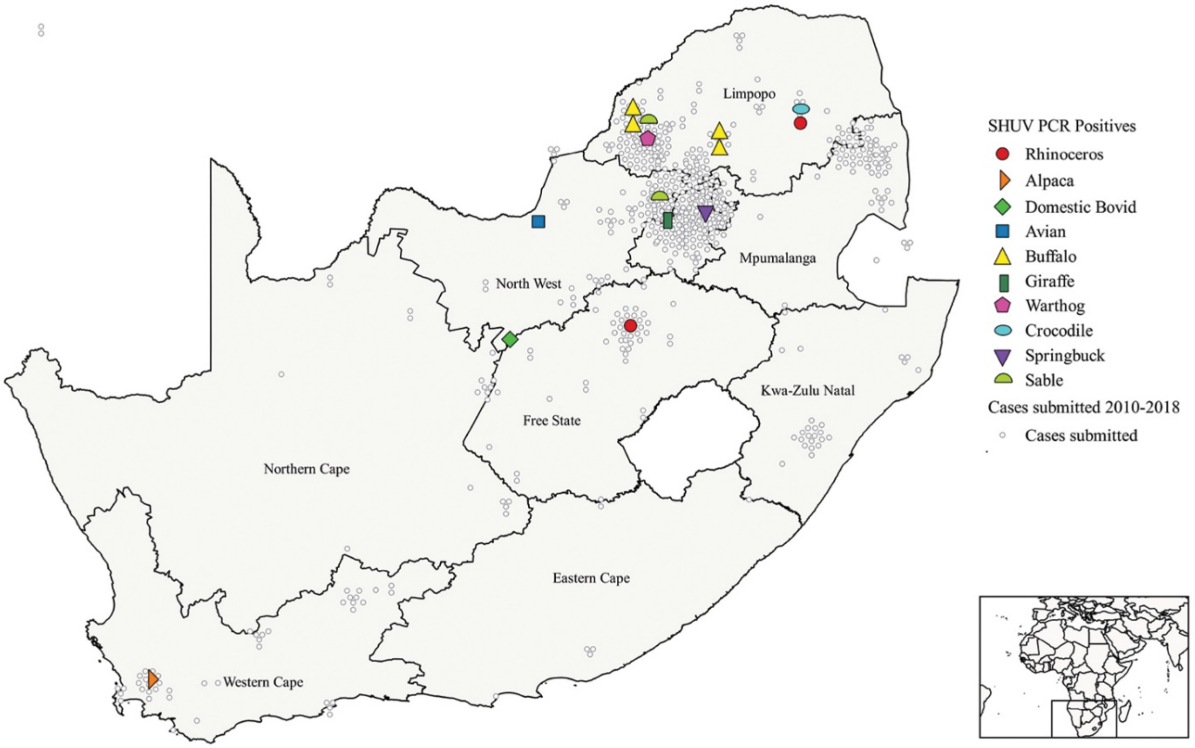

Appendix Figure 1. Map illustrating Shuni virus infections detected in wildlife, non-equine domestic animals and birds in South Africa from 2010 to 2018.

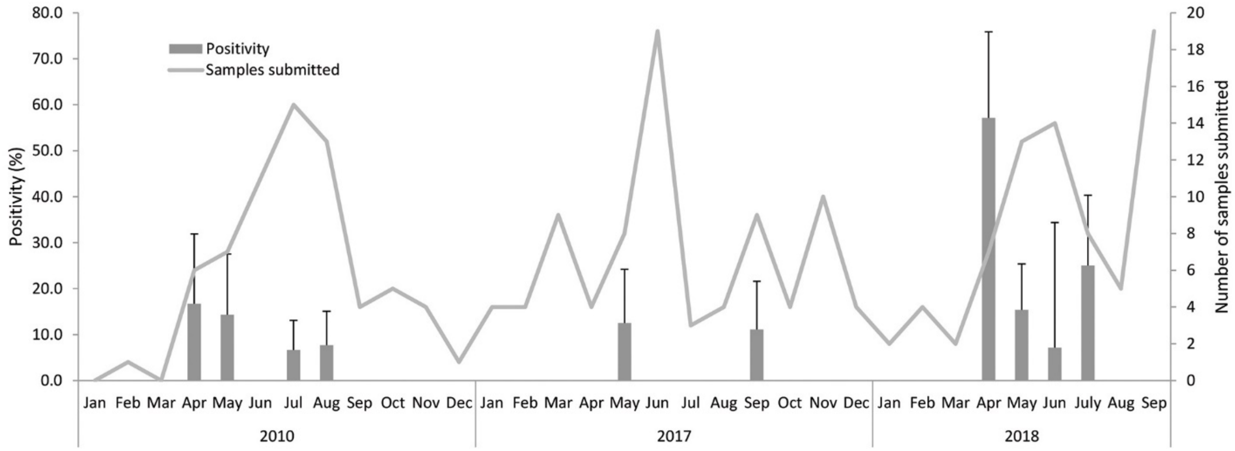

Appendix Figure 2. Seasonality of Shuni virus in wildlife and domestic animals during 2010–2018 in South Africa. Light gray bars indicate RT-PCR positives; line graph indicates samples submitted.

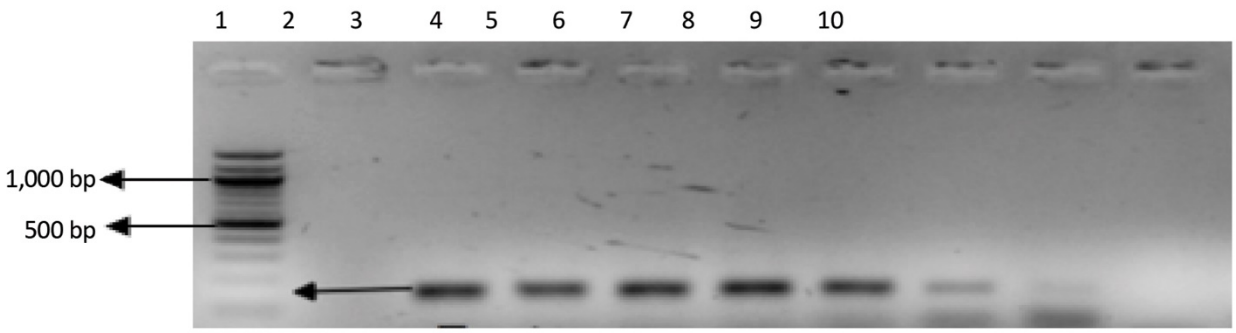

Appendix Figure 3. Agarose gel electrophoresis image showing Simbu/orthubunyavirus genus specific real-time TaqMan RT-PCR assay products separated on a 2.0% agarose gel. The band sizes were determined using a 100 bp molecular marker with an expected band size of 152 bp. Dilutions were made using the SHUV control (SAE18/09) and are displayed from the highest dilution (10^0) to the lowest (10^{-6}) (lanes 3–8) and a negative no-template control (lane 10).
